# Transparency and cooperation in repeated dilemma games: a meta study

**DOI:** 10.1007/s10683-017-9517-4

**Published:** 2017-02-24

**Authors:** Lenka Fiala, Sigrid Suetens

**Affiliations:** CentER, TILEC, Tilburg University, Tilburg, The Netherlands

**Keywords:** Information, Cooperation, Repeated game, Metastudy, Laboratory experiment, D8, H4, L1

## Abstract

We use data from experiments on finitely repeated dilemma games with fixed matching to investigate the effect of different types of information on cooperation. The data come from 71 studies using the voluntary contributions paradigm, covering 122 data points, and from 18 studies on decision-making in oligopoly, covering another 50 data points. We find similar effects in the two sets of experimental games. We find that transparency about what everyone in a group earns reduces contributions to the public good, as well as the degree of collusion in oligopoly markets. In contrast, transparency about choices tends to lead to an increase in contributions and collusion, although the size of this effect varies somewhat between the two settings. Our results are potentially useful for policy making, because they provide guidance on the type of information to target in order to stimulate or limit cooperation.

**Electronic supplementary material:**

The online version of this article (doi:10.1007/s10683-017-9517-4) contains supplementary material, which is available to authorized users.

## Introduction

Part of the social sciences is concerned with identifying determinants of voluntary cooperation in repeated group interactions, with the ultimate goal of obtaining knowledge about how cooperation can be influenced. How can individuals in a group be stimulated to contribute to a public good? But also, how can it be avoided that firms with market power collude? Clearly, the two environments in these examples—public good settings and oligopoly markets—are very different in many ways (for example, in terms of decision-makers and social-welfare effects of cooperation). However, the two settings also have a crucial feature in common; both have a ‘dilemma’ structure. In both settings, short-sighted selfishness of the players in the group leads to suboptimal outcomes for the group.^1^ The point is that the basic strategic nature of the decision problem of firms in a cartel is the same as that of the ‘free-rider’ problem. Therefore, when it comes to the very basics of behavior, we believe there is scope for cross-fertilization between the two fields.

An important determinant of behavior in finitely repeated dilemma games with fixed matching that has received considerable attention in both fields, is transparency about past outcomes or choices. Transparency can potentially be influenced by those who are in power to ‘govern groups’, and can therefore be an attractive tool to stimulate or push back cooperation.^2^ This tool can find use both on a macro scale (e.g., regulation in the form of forced disclosure), micro scale (e.g., incentives within companies), and in laboratory experiments, as an inconspicuous mechanism to induce certain behaviors. To apply transparency effectively, however, we must understand how different forms of it affect behavior, and whether the effects are general, or, instead, can be overridden by context or group size. This is exactly what we study.

Several ways have been identified through which transparency may increase cooperation in a group. First, giving players information about past choices made by others in the group makes it easier to spot deviations, and knowing whether a deviation has occurred is a necessary condition for stable long-run cooperation to be sustainable, at least in theory (Friedman 1971; Fudenberg and Maskin 1986; Benoit and Krishna 1984). Second, transparency allows cooperative players to signal their cooperative intentions, which may spur them to induce cooperation, and conditional cooperators to follow or imitate the induced cooperation (Fischbacher et al. 2001; Davis et al. 2010). Transparency may also impede cooperation, however. For one, if players receive information about the details of past choices and earnings, this may lead to an ‘imitation spiral’ where players imitate the most successful player, that is, the player who earned the highest amount, up until cooperation is completely destroyed (Vega-Redondo 1997; Huck et al. 2000). Also, receiving detailed information about earnings may put players in a relatively selfish state of mind, leading them to focus on the payoff consequences of their choices (Nikiforakis 2010). Finally, if detailed information about choices is provided, players may become aware of the potentially very unequal distribution of outcomes associated with it, and be less motivated to cooperate (Cheung 2014).

The current paper is set out to study similarities and differences across the two fields in how transparency about past choices and payoffs affects choices (contributions, prices, or quantities). The focus is on games with finitely repeated interaction, where the same group of (more than two) players repeatedly plays a dilemma game.^3^ These games can be taken to be representative for fundamentals of behavior of symmetric or close to symmetric agents, making decisions in relatively small groups (e.g., firms of similar size competing in oligopolistic industries, individuals working in teams, pollution problems in similar-sized countries). To do so, we assembled data from laboratory experiments on linear public goods games and oligopoly games of price- and quantity setting. The data set covers data from 71 studies on public goods, covering 122 different treatments that have 1205 independent observations and 5565 participants in total, and from 18 studies on decision-making in oligopoly, covering another 50 different treatments with 387 independent observations and 1339 participants.

Related meta-studies are those of Zelmer (2003) on linear public goods experiments and Engel (2007) on oligopoly experiments.^4^ Our study differs from these studies in that we focus on a specific topic—the effect of transparency about choices and payoffs on cooperation—and we study it across disciplines. Also, none of these studies track the details we track about the nature of feedback provided to participants in the experiments.

Our main finding is that transparency about past individual payoffs of group members is destructive for cooperation in both dilemma settings. In public goods experiments, this type of transparency leads to a significant reduction in contributions, whereas in oligopoly experiments, it leads to a significantly lower degree of collusion.

## The data

We include data from all laboratory experiments we could find where participants play a linear public goods game or an oligopoly game fulfilling the following conditions: (1) the experiment is incentivized, (2) participants play a finitely repeated game (partner or fixed matching), (3) the group size is larger than two, (4) the game is symmetric (same endowments, same costs, etc.), (5) decision-making is simultaneous, (6) the subgame perfect Nash equilibrium is unique and Pareto-dominated, (7) buyers (in the case of oligopoly experiments) are computerized, (8) communication or chat is not allowed, (9) the game or treatment is not (reported to be) preceded by another related game or treatment,^5^ and (10) the study is published in a scientific journal.

For all experiments, we recorded several variables by treatment. Hence, the unit of observation in our data set is the treatment level, and the total number of data points in the meta-analysis is equal to the number of studies times the number of treatments in each study.^6^ In what follows, we give a detailed description of the methodology we followed to collect the data of the public goods and oligopoly experiments.

### Public goods experiments

In a first phase, we collected studies searched for on Scopus in November 2012 using different combinations of keywords [experiment(s), voluntary contribution(s), public goods, linear, laboratory, subjects, participants], and the studies listed in the meta-study of Zelmer (2003). We restricted the search to published studies in English language, that could be accessed online via the library of Tilburg University. We also sent around private e-mails to authors of collected studies asking for information on certain variables, for cases where such information was difficult to extract from the study.

In a second phase, in February 2015, we sent around the references of the collected studies to the ESA Google Groups mailing list for experimental methods discussion. In the accompanying message, we asked to send us references of studies reporting on treatments fulfilling the above conditions and not included in the list.

In a third phase, we included three more studies that were missing, of which two were listed in Ledyard (1995). We ended up with 71 studies covering 122 treatments. Section 1 of the electronic supplementary material gives an overview of the collected studies, including an overview of the available data on the nature of feedback.

We recorded the initial endowment and the average contributions across all rounds and contributions in the first round as a percentage of the initial endowment, whenever possible, as well as the following range of binary variables that refer to the feedback participants get after each period of play: aggregate feedback about choices in one’s group (F.aggchoice), feedback about each group member’s choice (F.indichoice), feedback about each group member’s payoff (F.indipayoffs), and feedback about one’s own payoff (F.ownpayoff).^7^ We also recorded a range of control variables, including group size and the marginal per capita return of the public good (MPCR). MPCR and group size have been shown to increase contributions (see Isaac et al. 1994). Our dependent variable in the analysis based on the public goods data is the average contribution as a percentage of the initial endowment.

### Oligopoly experiments

As with the public goods experiments, we only used data from published studies in English language that could be accessed online via the library of Tilburg University. In a first phase, we collected the studies listed in the meta-study of Engel (2007), and studies covering oligopoly experiments that fulfilled the above-mentioned conditions listed in the references of Potters and Suetens (2013). We also sent around private e-mails to authors of collected studies asking for information on certain variables, for cases where such information was difficult to extract from the study.

In a second phase we used the ESA Google Groups mailing list for experimental methods discussion to ask for references reporting on treatments that fulfill a range of conditions, but that were not included in our list collected in the first phase.

In a third phase, we included three more studies that were listed in Engel (2015). We ended up with 18 studies covering 50 treatments. Section 2 of the electronic supplementary material gives an overview of the collected studies, including an overview of the available data on the nature of feedback.

We recorded the average choices across all rounds and the choice in the first round, as well as the same range of feedback dummy variables as in the public goods experiments. In addition, we recorded a range of theoretical benchmarks, including the SPNE choice, the (symmetric) joint-payoff-maximizing choice, and the ‘deviation’ payoff (payoff one receives if one best-responds to the rest of the group fully cooperating).^8^ If these theoretical benchmarks were not given, we calculated them ourselves using the parameters given. Out of the theoretical benchmarks, we composed the following index that serves as a measure for the scope for tacit collusion in repeated games along the lines of Friedman (1971): $\frac{\text{joint-payoff-maximizing payoff} - \text{SPNE payoff}}{\text{deviation payoff} - \text{joint-payoff-maximizing payoff}}$. We refer to this index as the ‘Friedman’ index, and expected it would have a positive effect on collusion.^9^ We also recorded group size and the nature of the strategic environment (strategic complements versus substitutes). We expected more competition (higher output and lower prices) in large groups (see Huck et al. 2004) and more collusion in games with strategic complements than in games with strategic substitutes (see Embrey et al. 2015; Engel 2007; Potters and Suetens 2009; Suetens and Potters 2007).

Our dependent variable in the analysis based on the oligopoly data is the degree of collusion, defined as the average choice minus the SPNE choice divided by the joint-payoff-maximizing choice minus the SPNE choice.^10^ The degree of collusion measures the extent to which participants deviate from the SPNE in the direction of joint-payoff maximization. To illustrate, if under Bertrand price competition the average price is higher (lower) than the SPNE price, then the degree of collusion is larger (smaller) than zero. In contrast, under Cournot quantity competition, an average quantity higher (lower) than the SPNE quantity, implies a degree of collusion below (above) zero. Our independent variables in both analyses are the feedback variables and the above-listed control variables.

### Descriptives

Table 1 provides summary statistics of the dependent and independent variables, and of average numbers of independent observations (groups or markets) and participants by data point. To illustrate, public goods experiments have on average about 10 independent observations in their treatments, and oligopoly experiments have on average 7.7. The average number of participants by treatment is 46 in public goods experiments and 27 in oligopoly experiments. The table further shows that both samples have variation in all feedback variables. We should further point out that the negative mean degree of collusion reported in the table may be attributed to our selection criterion that excludes two-player groups. In oligopolies with more than two players, behavior is typically quite rivalistic, especially under Cournot competition (e.g., Huck et al. 2004).

**Table 1:** Summary statistics

Variable	Public goods					Oligopoly				
	N	Mean	SD	Min.	Max.	N	Mean	SD	Min.	Max.
Share contributed	116	0.40	0.14	0.11	0.74					
Degree of collusion						50	−0.15	0.50	−2.40	0.90
F.indichoice	119	0.45	0.50	0	1	50	0.44	0.50	0	1
F.aggchoice	118	0.78	0.42	0	1	50	0.64	0.48	0	1
F.indipayoffs	119	0.11	0.31	0	1	50	0.28	0.45	0	1
F.ownpayoff	117	0.87	0.34	0	1	50	0.98	0.14	0	1
Group size	122	5.61	5.25	3	40	50	3.84	0.93	3	8
MPCR	122	0.46	0.14	0.03	0.75					
Strategic complements						50	0.26	0.44	0	1
‘Friedman’ index						46	0.83	0.55	0.13	3
# Indep. obs.	121	9.96	7.08	1	34	50	7.74	3.63	1	17
# Participants	121	45.99	29.32	4	136	50	26.78	9.88	5	48
# Periods	122	12.29	6.32	4	50	50	42.94	28.12	12	120

The table reports summary statistics. Variables that have a minimum of 0 and a maximum of 1 are binary variables. F.aggchoice refers to aggregate feedback about choices in one’s group, F.indichoice to feedback about each group member’s choice, F.indipayoffs to feedback about each group member’s payoff, and F.ownpayoff to feedback about one’s own payoff

**Table 2:** Cooperation depending on feedback

	F.indichoice		F.aggchoice		F.indipayoffs		F.ownpayoff	
	Yes	No	Yes	No	Yes	No	Yes	No
Average share contributed (all)	0.41	0.39	0.40	0.41	0.35	0.41	0.40	0.41
Average share contributed (MPCR < 0.7)	0.40	0.38	0.39	0.39	0.28	0.40	0.38	0.41
Average degree of collusion	−0.19	−0.12	−0.09	−0.26	−0.53	−0.00	−0.15	−0.06

The table reports the average share of endowment contributed (for public goods experiments) and the average degree of collusion (for oligopoly experiment) depending on the particular feedback present. F.aggchoice refers to aggregate feedback about choices in one’s group, F.indichoice to feedback about each group member’s choice, F.indipayoffs to feedback about each group member’s payoff, and F.ownpayoff to feedback about one’s own payoff

**Table 3:** Correlations between feedback variables

	F.indichoice	F.aggchoice	F.indipayoffs
Public goods experiments			
F.aggchoice	−0.465		
	(0.000)		
F.indipayoffs	0.330	−0.401	
	(0.000)	(0.000)	
F.ownpayoff	0.092	0.110	0.136
	(0.323)	(0.238)	(0.145)
Oligopoly experiments			
F.aggchoice	−0.678		
	(0.000)		
F.indipayoffs	0.704	−0.368	
	(0.000)	(0.009)	
F.ownpayoff	0.127	−0.107	0.089
	(0.381)	(0.459)	(0.538)

The table reports the correlation coefficients for each pair of feedback variables based on the data used in Tables 4 and 6. *p* values are in parentheses. F.aggchoice refers to aggregate feedback about choices in one’s group, F.indichoice to feedback about each group member’s choice, F.indipayoffs to feedback about each group member’s payoff, and F.ownpayoff to feedback about one’s own payoff

Table 2 gives a first impression of the effect of feedback on cooperation. It provides summary statistics on cooperation—the average share of the endowment contributed for public goods experiments and the average degree of collusion for oligopoly experiments—depending on the nature of feedback. For the public goods data, we also show averages across all experiments where the MPCR is lower than 0.7. The reason why we report the latter results is that few experiments implement an MPCR larger than 0.7 (see Sect. 3.1 for further motivation). The table shows that feedback about individual payoffs (F.indipayoffs) has the largest effect on cooperation in both sets of experiments, and the effect is negative. In the public goods data, the effect is particularly large for the games with an MPCR lower than 0.7. To illustrate, the share of the endowment individuals contribute when they have feedback about the payoffs is overall 6 percentage points lower than when they do not have such feedback. In the oligopoly games the difference in degree of collusion equals 0.53 points (notice that these points do not correspond to percentage points). The effects of the other feedback variables is less visible in the table, except that feedback about the aggregate choice seems to substantially increase the degree of collusion in the oligopoly experiments, whereas feedback about individual choices rather decreases it. We return to this finding later in the paper.

Finally, Table 3 reports pairwise correlations between the different feedback variables. As can be seen, some of the feedback variables are highly correlated. For example, F.indichoice and F.indipayoffs are positively and significantly correlated. It is important to be aware of these correlations for the interpretations of some of the regression results reported in Sect. 3.

## Statistical analysis

### Main results

Tables 4 and 5 report results from regressions based on the public goods data. Table 4 is based on all data and Table 5 on data from experiments where the MPCR is smaller than 0.7. As can be seen in the table, only 12 data points drop out, implying that in 12 of the data points the MPCR is above or equal to 0.7. All reported regression results are based on regressions that use numbers of independent observations by treatment as weights.^11^ Both tables have results from regressions that include the feedback variables one by one [specifications (1) to (4)], from a regression that includes all feedback variables at once without controlling for other effects [specification (5)], and from regressions that include all feedback variables controlling for other variables [specifications (6) to (8)].^12^

**Table 4:** Regression results public goods

	(1)	(2)	(3)	(4)	(5)	(6)	(7)	(8)
Dep. var.: Share of endowment contributed								
F.indichoice	0.030				0.042	0.056**	0.046	0.061**
	(0.257)				(0.149)	(0.044)	(0.123)	(0.032)
F.aggchoice		−0.007			−0.014	−0.015	−0.015	−0.016
		(0.835)			(0.723)	(0.697)	(0.707)	(0.676)
F.indipayoffs			−0.048		−0.073	−0.120**	−0.075	−0.123**
			(0.267)		(0.143)	(0.013)	(0.136)	(0.012)
F.ownpayoff				−0.002	0.004	0.032	0.010	0.039
				(0.974)	(0.927)	(0.482)	(0.846)	(0.404)
MPCR						0.446***		0.450***
						(0.000)		(0.000)
Group size							0.004	0.004
							(0.478)	(0.353)
Constant	0.404***	0.426***	0.425***	0.419***	0.411***	0.174**	0.389***	0.145*
	(0.000)	(0.000)	(0.000)	(0.000)	(0.000)	(0.025)	(0.000)	(0.082)
$R^2$	0.011	0.000	0.011	0.000	0.035	0.165	0.040	0.172
Adj. $R^2$	0.003	−0.009	0.002	−0.009	−0.001	0.126	−0.005	0.125
* N*	116	115	116	114	113	113	113	113

The table reports results from linear regressions based on data from public goods experiments. The numbers of independent observations by unit of observation (treatment) are used as weights (*p* values in parentheses). Stars ***, ** or * indicate that the effect of the variable is statistically significant at the 1, 5 or 10% level, respectively. F.aggchoice refers to aggregate feedback about choices in one’s group, F.indichoice to feedback about each group member’s choice, F.indipayoffs to feedback about each group member’s payoff, and F.ownpayoff to feedback about one’s own payoff

**Table 5:** Regression results public goods with MPCR < 0.7

	(1)	(2)	(3)	(4)	(5)	(6)	(7)	(8)
Dep. var.: Share of endowment contributed								
F.indichoice	0.024				0.051*	0.053*	0.054*	0.057*
	(0.397)				(0.083)	(0.070)	(0.072)	(0.054)
F.aggchoice		0.028			0.019	0.006	0.018	0.004
		(0.457)			(0.638)	(0.892)	(0.649)	(0.922)
F.indipayoffs			−0.173***		−0.181***	−0.180***	−0.182***	−0.181***
			(0.001)		(0.001)	(0.001)	(0.001)	(0.001)
F.ownpayoff				−0.015	−0.009	0.012	−0.004	0.020
				(0.764)	(0.803)	(0.808)	(0.927)	(0.691)
MPCR						0.300*		0.317*
						(0.070)		(0.058)
Group size							0.003	0.004
							(0.561)	(0.412)
Constant	0.397***	0.387***	0.421***	0.421***	0.386***	0.244**	0.367***	0.210**
	(0.000)	(0.000)	(0.000)	(0.000)	(0.000)	(0.011)	(0.000)	(0.045)
$R^2$	0.007	0.006	0.098	0.001	0.125	0.155	0.128	0.161
Adj. $R^2$	−0.003	−0.004	0.089	−0.009	0.088	0.110	0.082	0.107
*N*	104	103	104	102	101	101	101	101

The table reports results from linear regressions based on data from public goods experiments. The numbers of independent observations by unit of observation (treatment) are used as weights (*p* values in parentheses). Stars ***, ** or * indicate that the effect of the variable is statistically significant at the 1, 5 or 10 level, respectively. F.aggchoice refers to aggregate feedback about choices in one’s group, F.indichoice to feedback about each group member’s choice, F.indipayoffs to feedback about each group member’s payoff, and F.ownpayoff to feedback about one’s own payoff

First, we consider Table 4. As shown in specifications (1) to (5), none of the feedback variables have a statistically significant effect on the share of endowment contributed to the public good if no other variables are controlled for. In terms of magnitude, the effects of F.aggchoice and of F.ownpayoff are negligible. The effect of F.indichoice, and, in particular, that of F.indipayoffs, is larger in magnitude. If controlling for group size, as is done in specification (7), results are very similar because group size has no significant effect. If controlling for the MPCR, however, as is done in specifications (6) and (8), the positive effect of F.indichoice and the negative effect of F.indipayoffs have a larger magnitude and turn significant at the 5% level. The share contributed tends to be about 6 percentage points higher than in cases where players receive feedback about the distribution of choices in their group. Provision of feedback about individual payoffs tends to reduce the share contributed to the public good by about 12 percentage points. Also, in line with the expectations, the MPCR has a large, positive effect on contributions.

Next, we turn to Table 5, which reports results from regressions based on public goods games with an MPCR lower than 0.7.^13^ We include these results because there are few data points on public goods games with an MPCR higher than 0.7 (only 12). It happens to be the case that for these data points F.indichoice and F.indipayoffs are highly positively correlated.^14^ Consequently, including these data points may lead to an underestimation of the ‘strength’ of the negative effect of F.indipayoffs in ‘typical’ public goods games. Results related to feedback variables F.indichoice, F.aggchoice and F.ownpayoff are qualitatively very similar to those based on all data; F.aggchoice and F.ownpayoff have basically no effect and F.indichoice increases the percentage contribution by about 5–6 percentage points if controlling for other variables (significant at the 10% level). With respect to F.indipayoffs we observe that its effect becomes larger in magnitude and highly statistically significant even without controlling for MPCR. The marginal effect is now 17–18 percentage points. In addition, as shown in specifications (6) and (8), the effect of MPCR decreases somewhat in magnitude.

Next, we consider the results for the oligopoly games, which are presented in Table 6. The table has eight specifications depending on whether the feedback variables are included one by one, and depending on whether controls are included. There are three things that come to mind when looking at the table. The first is that F.indipayoffs has a negative and significant effect across all specifications, thereby leading to a drop in degree of collusion of 0.48 to 0.78 points, depending on the specification. Just like in the public goods experiments, transparency about individual payoffs of group members thus decreases cooperation. Second, F.indichoice has a negative (but not significant) effect on the degree of collusion when included on its own and has a positive and (mostly) highly significant effect when included together with F.indipayoffs. The reason behind this shift in estimate is that F.indichoice and F.indipayoffs are highly positively correlated (see Table 3); in many oligopoly experiments both pieces of feedback are provided. Therefore, if included on its own, F.indichoice picks up the negative effect of F.indipayoffs thereby producing a biased estimate. For the public goods data, a similar tendency is observed although that the effect of F.indichoice does not become negative when included on its own [compare specifications (5) to (8) to specification (1) in Tables 4 and 5]. A third result is that F.aggchoice has a significantly positive and substantial effect on the degree of collusion across all specifications. When players receive aggregate information about the past choices of group members, it thus seems that competition, which can be quite fierce in oligopoly settings in groups larger than two, seems to be substantially reduced. The marginal effect depends somewhat on the specification, but, overall, having aggregate information about choices of others increases the degree of collusion by about 0.30 to 0.40 points.

**Table 6:** Regression results oligopoly

	(1)	(2)	(3)	(4)	(5)	(6)	(7)	(8)
Dep. var.: Degree of collusion								
F.indichoice	−0.188				0.556***	0.499***	0.156	0.491***
	(0.131)				(0.003)	(0.003)	(0.428)	(0.004)
F.aggchoice		0.306**			0.335**	0.404***	0.358***	0.414***
		(0.015)			(0.016)	(0.002)	(0.005)	(0.002)
F.indipayoffs			−0.481***		−0.782***	−0.643***	−0.398**	−0.639***
			(0.000)		(0.000)	(0.000)	(0.027)	(0.000)
F.ownpayoff				−0.132	−0.026	−0.101	0.594	−0.063
				(0.790)	(0.947)	(0.780)	(0.192)	(0.862)
Complements						0.374***	0.459***	0.374***
						(0.002)	(0.000)	(0.002)
‘Friedman’ index							0.307**	
							(0.021)	
Group size								−0.053
								(0.358)
Constant	−0.117	−0.395***	−0.061	−0.060	−0.395	−0.464	−1.339**	−0.313
	(0.131)	(0.000)	(0.327)	(0.903)	(0.344)	(0.221)	(0.012)	(0.447)
$R^2$	0.047	0.116	0.255	0.001	0.403	0.522	0.567	0.532
Adj. $R^2$	0.027	0.098	0.239	−0.019	0.350	0.468	0.500	0.467
*N*	50	50	50	50	50	50	46	50

The table reports results from linear regressions based on data from oligopoly experiments. The numbers of independent observations by unit of observation (treatment) are used as weights (*p* values in parentheses). Stars ***, ** or * indicate that the effect of the variable is statistically significant at the 1, 5 or 10% level, respectively. F.aggchoice refers to aggregate feedback about choices in one’s group, F.indichoice to feedback about each group member’s choice, F.indipayoffs to feedback about each group member’s payoff, and F.ownpayoff to feedback about one’s own payoff

Regarding the control variables, in line with our expectations, the degree of collusion is higher in the case of strategic complementarity, and is positively related to the ‘Friedman’ index (both significant). Against the expectation, group size does not have a significant effect.

In summary, transparency about individual payoffs of group members leads to a substantial and significant decrease in cooperation in both dilemma settings. Transparency about individual choices tends to increase cooperation, but the effect is smaller in magnitude. Transparency about aggregate choices increases cooperation in oligopoly games, and not so in public goods games.

At first sight, it may seem remarkable that feedback about choices of group members in an aggregate format dramatically increases cooperation in oligopoly settings but not so in public good settings. We speculate that the differential effect may be due to the fact that, unlike in linear public goods games, in oligopoly settings there is scope for behaving more competitively than in the SPNE. Since ‘baseline’ behavior is typically more competitive than in the SPNE, particularly in settings with strategic substitutes (Engel 2007; Suetens and Potters 2007), there is more potential for transparency about choices to increase cooperation. Regressions based exclusively on games with strategic substitutes suggest that the effect of transparency about aggregate choices is indeed particularly large in these environments (based on 37 data points, not reported). In games with strategic complements, no such effect is observed.^15^

### Publication bias

The regression results reported so far are based on data stemming from, on the one hand, experiments that either implement one particular feedback setting, and, on the other hand, from experiments that explicitly test for the effect of feedback on behavior. The data set thus consists of data from (1) experiments with treatments that all have the same feedback conditions, and (2) experiments where feedback conditions vary between treatments within the experiment. In the current section, we investigate whether the earlier reported results are robust to removing data from treatments of studies that were explicitly designed to study the effect of feedback on behavior. It may be that due to a publication bias studies that generate significant effects (for example, a significant effect of transparency about earnings on collusion) are more likely to be published. If the above-reported results no longer hold after removal of data from experiments with variation in feedback, this would be an indication of publication bias.

**Table 7:** Regression results reduced sample

	Pg	Oligopoly	
	(1)	(2)	(3)
F.indichoice	0.062**	–	–
	(0.035)		
F.aggchoice	−0.006	−0.070	−0.359**
	(0.875)	(0.793)	(0.042)
F.indipayoffs	−0.114**	−0.584**	−0.861***
	(0.042)	(0.041)	(0.000)
F.ownpayoff	0.036	0.227	−0.114
	(0.453)	(0.750)	(0.571)
MPCR	0.450***		
	(0.000)		
Complements		0.228	0.187
		(0.139)	(0.197)
‘Friedman’ index		0.141	
		(0.636)	
Group size	0.004		0.013
	(0.421)		(0.725)
Constant	0.142*	−0.413	0.260
	(0.096)	(0.715)	(0.345)
$R^2$	0.163	0.823	0.800
Adj. $R^2$	0.113	0.774	0.755
*N*	107	24	28

The table reports results from linear regressions based on data from public goods [specification (1)] and oligopoly experiments [specifications (2) and (3)] that have no treatment variation in one of the feedback variables. The dependent variables are share of endowment contributed, and degree of collusion, respectively. The numbers of independent observations by unit of observation (treatment) are used as weights (*p* values in parentheses). Stars ***, ** or * indicate that the effect of the variable is statistically significant at the 1, 5 or 10% level, respectively. F.aggchoice refers to aggregate feedback about choices in one’s group, F.indichoice to feedback about each group member’s choice, F.indipayoffs to feedback about each group member’s payoff, and F.ownpayoff to feedback about one’s own payoff

Our approach is thus to remove those data from experiments that have within-treatment variation in one of the feedback variables, and to run the earlier-reported regressions on the reduced data set. Table 7 reports the results from regressions based on the public goods and oligopoly data. We report the specifications where all feedback variables and controls are included, and refer to Tables S4 and S5 in the Sect. 3 of the electronic supplementary material for other specifications. Because the public goods experiments typically do not vary feedback conditions between treatments, only few data drop out. This is in contrast to the oligopoly data, where less than half of the data points are left.

As can be seen in Table 7, the results for the public goods experiments are similar to the above-reported results. In the small sample of remaining oligopoly data [specifications (2) and (3)], the above-reported main result related to transparency about individual earnings carries through. This is reassuring because it suggests that this is a robust finding. The result related to feedback about aggregate choices does not carry through, however.

## Concluding remarks

This meta-study shows that transparency about individual payoffs of group members is destructive for cooperation in public goods games as well as in oligopoly games. The finding is well in accordance with the theoretical result by Vega-Redondo (1997) that if players imitate the most successful—least cooperative—player, a dynamic sets in that leads to a reduction in cooperation. If feedback about individual payoffs is available, players may be more led by such dynamic than when not available. In addition, such feedback may help players to focus on payoffs (Nikiforakis 2010), or make them more aware of the potentially very unequal payoff distributions (Cheung 2014), which may also lead them to behave more competitively.

Although we cannot discriminate between these three explanations, we can say something about the importance of dynamics. That is, we can study whether it is necessary that players experience the payoff feedback before its effect sets in. We find that merely knowing that one will receive detailed information about payoffs does not seem to be sufficient to affect cooperation. In particular, receiving feedback about individual earnings has no significant effect on contributions or degree of collusion in the very first period of play of a repeated game. To illustrate, in regressions with share of endowment contributed or degree of collusion in the first period as dependent variable and all feedback variables and controls as independent variables, we find that earnings feedback has no significant effect; the estimated effect is −0.0002 ($p=0.996$) in the public goods games, and −0.481 ($p=0.562$) in the oligopoly games. This suggests that dynamics are likely to be important for the effect to set in.^16^

We also find that when subjects are provided with information on the individual choices of other group members, cooperation increases in both dilemma settings. When subjects are provided with aggregate information about choices, cooperation only increases in the oligopoly setting. We speculate that this may be due to oligopoly, and in particular, Cournot environments, providing the possibility to behave more competitively than in the static equilibrium. In the public goods game, playing static-equilibrium choices is the most competitive possible behavior so there is less scope for cooperation to increase. In addition, we should note that the effects of feedback about choices are less robust. In particular, when removing studies specifically designed to study the effect of feedback, the estimated effects in the oligopoly games change.

That the causal effect of transparency about payoffs on cooperation generalizes to at least two fields lends support to its external validity. For those governing groups, the results suggest that they may use transparency about group members’ actions and payoffs as a tool to influence cooperation in the group. Transparency about individual payoffs is likely to push back cooperation whereas (with some reservations) transparency about actions may stimulate cooperation.

In the experiments included in the meta-study communication was not possible. The effect of payoff feedback may be different in environments where communication is possible. Remarkably, Gomez-Martinez et al. (2016) find in a Cournot experiment that transparency about payoffs leads to *more* collusion in an environment where explicit collusion is allowed by means of communication. It is an interesting question whether this is a robust finding, for example, whether their result also holds for public goods games.

Finally, the results from the meta-study are also of relevance for researchers designing public goods or oligopoly experiments. The choice of which feedback to provide may not be innocent.

## Electronic supplementary material

Below is the link to the electronic supplementary material.

Supplementary material 1 (pdf 109 KB)

Supplementary material 2 (pdf 90 KB)

Supplementary material 3 (pdf 101 KB)

## Footnotes

1 In the case of oligopoly, this holds, of course, only if the group refers to the firms in the market, excluding consumers.

2 See Potters (2009) for an overview of results from laboratory experiments on the effects of transparency on tacit collusion in oligopolistic markets, including other types of transparency than the ones we deal with (e.g., transparency about demand function).

3 In pairs of players the informational content of receiving feedback about the partner’s past choice or payoff is much lower than in groups of more than two players. Even if this information is not explicitly provided, it is easy to infer the partner’s choice and payoff from the information one receives about one’s own payoff or from aggregate information about choices. Therefore, we do not consider pairs.

4 Sally (1995) is another meta-study on prisoner's dilemma experiments. Croson and Marks (2000) is a meta-study on threshold public good games.

5 If a paper used a within-subject design, we only included data from the first treatment the subjects faced, provided that this treatment fit our other criteria.

6 For some experiments, only one treatment fulfills the above-mentioned conditions, so that only one treatment is included in the data set. For other experiments, data from more than one treatment are included.

7 We also recorded the variable F.othergroups, which indicates whether participants received feedback about choices in other groups. Given that this variable only has variation in the public goods sample and not so in the oligopoly sample, and that it did not have any effect on contributions, we excluded it from our analysis.

8 A higher such payoff gives higher incentives to deviate from potential tacit collusion.

9 The Friedman index is a negative function of group size. Therefore, in the regressions, we either control for the Friedman index or for group size.

10 We define the degree of collusion as $\frac{\text{average choice} - \text{SPNE choice}}{\text{joint-payoff-maximizing choice} - \text{SPNE choice}}$.

11 Results from unweighted regressions are qualitatively similar. See Tables S1 and S2 in Sect. 3 of the electronic supplementary material.

12 We report results related to F.ownpayoff for the sake of completeness. One may argue that including F.ownpayoff is not particularly meaningful because in practically all experiments information about own payoff is provided after each period of play. Removing F.ownpayoff from regressions (5) to (8) does not change any of the qualitative findings, though, nor does it have an effect on statistical significance of any of the other variables.

13 The cut-off level for the MPCR at 0.7 is, of course, arbitrary. Using a cut-off level of, e.g., 0.6 gives the same qualitative results.

14 To illustrate, the correlation is 0.8165 based on data where MPCR $\ge$ 0.7 versus 0.2785 based on data where MPCR < 0.7.

15 A caveat is that for games with strategic complements, we only have 13 data points, which may be too small to lead to meaningful conclusions. Note also that the results of the regressions mentioned in this paragraph are available upon request.

16 In a different exercise we checked whether earnings feedback has a stronger effect in relatively long games as compared to relatively short games after dividing both samples into short and long games using a median split. In the public goods games results are very similar for short and long games. In the oligopoly games there is a qualitatively (but statistically not significantly) stronger effect of earnings feedback in long games than in short games. However, there may be an effect from length of game that works in the opposite direction than earnings feedback, namely that of reputation building along the lines of Kreps et al. (1982).
