# Supplementary material for: Transparency and cooperation in repeated dilemma games: a meta study
Source: Exp Econ. 2017 Feb 24;20(4):755–71. doi: 10.1007/s10683-017-9517-4 (PMC5665965; doi:10.1007/s10683-017-9517-4)
Supplement: Supplementary file 1 — Supplementary material 1 (pdf 109 KB) [file 10683_2017_9517_MOESM1_ESM.pdf]

S1 Included Studies Public Goods Experiments

Article	F.aggchoice	F.indichoice	F.indipayoff	F.ownpayoff
Anderson and Mellor (2009)		X		n.a.
Andreoni (1988)	X			X
Bigoni and Suetens (2012)	X	Y	Y	X
Bochet and Putterman (2009)		X		
Bochet et al. (2006)		X		X
Bougherara et al. (2011)		X		X
Brañas–Garza and Espinosa (2011)				X
Cardenas et al. (2011)	X			X
Carpenter et al. (2004)	X	X		X
Castro (2008)	X			X
Chaudhuri et al. (2006)	X			X
Chun et al. (2011)	X			
Cinyabuguma et al. (2005)		X	X	X
Croson (1995)	X	X		X
Croson (1996)	X			X
Croson (2000)	X			X
Croson et al. (2005)		X		X
Drouvelis and Jamison (2012)	X			X
Eckel et al. (2015)	X			X
Fatas et al. (2010)	X	X		X
Ferraro and Vossler (2010)	X			X
Fehr and Gächter (2000)	X			X
Fuster and Meier (2010)	X			X
Gächter and Fehr (1999)	X			
Gächter et al. (2008)	X	X		X
Gonzalez et al. (2005)	X			X
Haigner and Wakolbinger (2010)		X		
Hamman et al. (2011)	X	X		X
Herrmann et al. (2008)	X	X		X
Houser and Kurzban (2002)	X	X		X
Isaac and Walker (1988a)	X			X
Isaac and Walker (1988b)	X			X
Isaac and Walker (1998)	X			X
Isaac et al. (1984)	X			X
Isaac et al. (1994)	X			X

Article	F. aggchoice	F. indichoice	F. indipayoffs	F. own payoff
Keser and van Winden (2000)	X			
Khadjavi and Lange (2011)	X			X
Kinukawa et al.(2000)	X			X
Koukoumelis et al. (2012)		X		X
Kroll et al. (2007)	n.a.	n.a.	n.a.	n.a.
Kurzban (2001)	X			X
Laury et al. (1995)	X			X
Leibbrandt et al. (2014)	X	X		
Lopez et al. (2012)		X		
Maier-Rigaud et al. (2010)		X		X
Markussen et al. (2014)		X		X
Neugebauer et al. (2009)		Y		Y
Nosenzo et al. (2013)		X	X	X
Ockenfels and Weimann (1999)	X			X
Ottone and Ponzano (2010)	n.a	n.a	n.a.	n.a.
Page et al. (2005)	n.a.	X		X
Peeters and Vorsatz (2013)	X	X		X
Putterman et al. (2011)	n.a.	n.a.	n.a.	n.a.
Qin et al. (2011)	X			
Reuben and Riedl (2013)		X	X	X
Rivas and Sutter (2011)		X		X
Saijo and Nakamura (1995)	X			
Sauermann and Glassmann (2014)	X	X		X
Sausgruber (2009)	X			X
Savikhin and Sheremeta (2013)	X			X
Sefton et al. (2007)	X	X		X
Sell and Wilson (1991)	Y	Y		Y
Shanley and Grossman (2007)	X			n.a.
Solow and Kirkwood (2002)	X			X
Stoddard (2014)	X			X
Stoddard et al. (2014)	X			X
Sutter et al. (2010)		X		X
Van Soest et al. (2016)		X		X
Vyrastekova et al. (2011)	X	X	X	X
Weimann (1994)	Y	Y	Y	X
Xiao and Houser (2011)		X		X

Notes: X means used in all treatments, Y means used in some treatments only, no symbol means feedback not given, and n.a. means data is missing. F.aggchoice refers to aggregate feedback about choices in one's group, F.indichoice to feedback about each group member's choice, F.indipayoffs to feedback about each group member's payoff, and F.ownpayoff to feedback about one's own payoff.

List of Public Goods Studies

Anderson, L. R., & Mellor, J. M. (2009). Religion and cooperation in a public goods experiment. *Economics Letters*, *105*(1), 58–60.

Andreoni, J. (1988). Why free ride?: Strategies and learning in public goods experiments. *Journal of Public Economics*, *37*(3), 291–304.

Bigoni, M., & Suetens, S. (2012). Feedback and dynamics in public good experiments. *Journal of Economic Behavior & Organization*, *82*(1), 86–95.

Bochet, O., Page, T., & Putterman, L. (2006). Communication and punishment in voluntary contribution experiments. *Journal of Economic Behavior & Organization*, *60*(1), 11–26.

Bochet, O., & Putterman, L. (2009). Not just babble: Opening the black box of communication in a voluntary contribution experiment. *European Economic Review*, *53*(3), 309–326.

Bougherara, D., Denant-Boemont, L., & Masclet, D. (2011). Cooperation and framing effects in provision point mechanisms: Experimental evidence. *Ecological Economics*, *70*(6), 1200–1210.

Brañas-Garza, P., & Espinosa, M. P. (2011). Unraveling public good games. *Games*, *2*(4), 434–451.

Cárdenas, J. C., Rodriguez, L. A., & Johnson, N. (2011). Collective action for watershed management: field experiments in Colombia and Kenya. *Environment and Development Economics*, *16*(03), 275–303.

Carpenter, J. P., Daniere, A. G., & Takahashi, L. M. (2004). Cooperation, trust, and social capital in Southeast Asian urban slums. *Journal of Economic Behavior & Organization*, *55*(4), 533–551.

Castro, M. F. (2008). Where are you from? Cultural differences in public good experiments. *The Journal of Socio-Economics*, *37*(6), 2319–2329.

Chaudhuri, A., Graziano, S., & Maitra, P. (2006). Social learning and norms in a public goods experiment with inter-generational advice. *The Review of Economic Studies*, *73*(2), 357–380.

Chun, Y., Kim, J., & Saijo, T. (2011). The spite dilemma experiment in Korea. *Seoul Journal of Economics*, *24*(1), 87.

Cinyabuguma, M., Page, T., & Putterman, L. (2005). Cooperation under the threat of expulsion in a public goods experiment. *Journal of Public Economics*, *89*(8), 1421–1435.

Croson, R., Fatas, E., & Neugebauer, T. (2005). Reciprocity, matching and conditional cooperation in two public goods games. *Economics Letters*, *87*(1), 95–101.

Croson, R. T. (1995). *Feedback in voluntary contribution mechanisms: an experiment in team production.* Wharton Risk Management and Decision Processes Center.

Croson, R. T. (1996). Partners and strangers revisited. *Economics Letters*, *53*(1), 25–32.

Croson, R. T. (2000). Thinking like a game theorist: factors affecting the frequency of equilibrium play. *Journal of Economic Behavior & Organization*, *41*(3), 299–314.

Drouvelis, M., & Jamison, J. C. (2015). Selecting public goods institutions: Who likes to punish and reward? *Southern Economic Journal*, *82*(2), 501–534.

Eckel, C. C., & Harwell, H. (2015). Four classic public goods experiments: A replication study. *Replication in Experimental Economics*, *18*, 13.

Fatas, E., Morales, A. J., & Ubeda, P. (2010). Blind justice: An experimental analysis of random punishment in team production. *Journal of Economic Psychology*, *31*(3), 358–373.

Fehr, E., & Gächter, S. (2000). Cooperation and punishment in public goods experiments. *American Economic Review*, *90*(4), 980–994.

Ferraro, P. J., & Vossler, C. A. (2010). The source and significance of confusion in public goods experiments. *The BE Journal of Economic Analysis & Policy*, *10*(1).

Fuster, A., & Meier, S. (2010). Another hidden cost of incentives: The detrimental effect on norm enforcement. *Management Science*, *56*(1), 57–70.

Gächter, S., & Fehr, E. (1999). Collective action as a social exchange. *Journal of Economic Behavior & Organization*, *39*(4), 341–369.

Gächter, S., Renner, E., & Sefton, M. (2008). The long-run benefits of punishment. *Science*, *322*(5907), 1510–1510.

Gonzalez, L. G., Güth, W., & Levati, M. V. (2005). When does the game end? Public goods experiments with non-definite and non-commonly known time horizons. *Economics Letters*, *88*(2), 221–226.

Haigner, S. D., & Wakolbinger, F. (2010). To lead or not to lead: Endogenous sequencing in public goods games. *Economics Letters*, *108*(1), 93–95.

Hamman, J. R., Weber, R. A., & Woon, J. (2011). An experimental investigation of electoral delegation and the provision of public goods. *American Journal of Political Science*, *55*(4), 738–752.

Herrmann, B., Thöni, C., & Gächter, S. (2008). Antisocial punishment across societies. *Science*, *319*(5868), 1362–1367.

Houser, D., & Kurzban, R. (2002). Revisiting kindness and confusion in public goods experiments. *The American Economic Review*, *92*(4), 1062–1069.

Isaac, R. M., & Walker, J. M. (1988a). Communication and free-riding behavior: The voluntary contribution mechanism. *Economic Inquiry*, *26*(4), 585–608.

Isaac, R. M., & Walker, J. M. (1988b). Group size effects in public goods provision: The voluntary contributions mechanism. *The Quarterly Journal of Economics*, 179–199.

Isaac, R. M., & Walker, J. M. (1998). Nash as an organizing principle in the voluntary provision of public goods: Experimental evidence. *Experimental Economics*, *1*(3), 191–206.

Isaac, R. M., Walker, J. M., & Thomas, S. H. (1984). Divergent evidence on free riding: An experimental examination of possible explanations. *Public Choice*, *43*(2), 113–149.

Isaac, R. M., Walker, J. M., & Williams, A. W. (1994). Group size and the voluntary provision of public goods: experimental evidence utilizing large groups. *Journal of Public Economics*, *54*(1), 1–36.

Keser, C., & Van Winden, F. (2000). Conditional cooperation and voluntary contributions to public goods. *The Scandinavian Journal of Economics*, *102*(1), 23–39.

Khadjavi, M., & Lange, A. (2015). Doing good or doing harm: experimental evidence on giving and taking in public good games. *Experimental Economics*, *18*(3), 432–441.

Kinukawa, S., Saijo, T., & Une, M. (2000). Partial communication in a voluntary-contribution-mechanism experiment. *Pacific Economic Review*, *5*(3), 411–428.

Koukoumelis, A., Levati, M. V., & Weisser, J. (2012). Leading by words: A voluntary contribution experiment with one-way communication. *Journal of Economic Behavior & Organization*, *81*(2), 379–390.

Kroll, S., Cherry, T. L., & Shogren, J. F. (2007). Voting, punishment, and public goods. *Economic Inquiry*, *45*(3), 557–570.

Kurzban, R. (2001). The social psychophysics of cooperation: Nonverbal communication in a public goods game. *Journal of Nonverbal Behavior*, *25*(4), 241–259.

Laury, S. K., Walker, J. M., & Williams, A. W. (1995). Anonymity and the voluntary provision of public goods. *Journal of Economic Behavior & Organization*, *27*(3), 365–380.

Leibbrandt, A., Ramalingam, A., Sääksvuori, L., & Walker, J. M. (2015). Incomplete punishment networks in public goods games: experimental evidence. *Experimental Economics*, *18*(1), 15–37.

Lopez, M. C., Murphy, J. J., Spraggon, J. M., & Stranlund, J. K. (2012). Comparing the effectiveness of regulation and pro-social emotions to enhance cooperation: Experimental evidence from fishing communities in Colombia. *Economic Inquiry*, *50*(1), 131–142.

Maier-Rigaud, F. P., Martinsson, P., & Staffiero, G. (2010). Ostracism and the provision of a public good: experimental evidence. *Journal of Economic Behavior & Organization*, *73*(3), 387–395.

Markussen, T., Reuben, E., & Tyran, J.-R. (2014). Competition, cooperation and collective choice. *The Economic Journal*, *124*(574), F163–F195.

Neugebauer, T., Perote, J., Schmidt, U., & Loos, M. (2009). Selfish-biased conditional cooperation: On the decline of contributions in repeated public goods experiments. *Journal of Economic Psychology*, *30*(1), 52–60.

Nosenzo, D., Quercia, S., & Sefton, M. (2015). Cooperation in small groups: The effect of group size. *Experimental Economics*, *18*(1), 4–14.

Ockenfels, A., & Weimann, J. (1999). Types and patterns: an experimental East-West-German comparison of cooperation and solidarity. *Journal of Public Economics*, *71*(2), 275–287.

Ottone, S., & Ponzano, F. (2010). Competition and cooperation in markets. The experimental case of a winner-take-all setting. *The Journal of Socio-Economics*, *39*(2), 163–170.

Page, T., Putterman, L., & Unel, B. (2005). Voluntary association in public goods experiments: Reciprocity, mimicry and efficiency*. *The Economic Journal*, *115*(506), 1032–1053.

Peeters, R., & Vorsatz, M. (2013). Immaterial rewards and sanctions in a voluntary contribution experiment. *Economic Inquiry*, *51*(2), 1442–1456.

Putterman, L., Tyran, J.-R., & Kamei, K. (2011). Public goods and voting on formal sanction schemes. *Journal of Public Economics*, *95*(9), 1213–1222.

Qin, X., Shen, J., & Meng, X. (2011). Group-based trust, trustworthiness and voluntary cooperation: Evidence from experimental and survey data in China. *The Journal of Socio-Economics*, *40*(4), 356–363.

Reuben, E., & Riedl, A. (2013). Enforcement of contribution norms in public good games with heterogeneous populations. *Games and Economic Behavior*, *77*(1), 122–137.

Rivas, M. F., & Sutter, M. (2011). The benefits of voluntary leadership in experimental public goods games. *Economics Letters*, *112*(2), 176–178.

Saijo, T., & Nakamura, H. (1995). The spite dilemma in voluntary contribution mechanism experiments. *Journal of Conflict Resolution*, *39*(3), 535–560.

Sauermann, J., & Glassmann, U. (2014). Restraining free-riders: The effects of actor types and decision rules in the public goods game. *Rationality and Society*, *26*(3), 290–319.

Sausgruber, R. (2009). A note on peer effects between teams. *Experimental Economics*, *12*(2), 193–201.

Savikhin, A. C., & Sheremeta, R. M. (2013). Simultaneous decision-making in competitive and cooperative environments. *Economic Inquiry*, *51*(2), 1311–1323.

Sefton, M., Shupp, R., & Walker, J. M. (2007). The effect of rewards and sanctions in provision of public goods. *Economic Inquiry*, *45*(4), 671–690.

Sell, J., & Wilson, R. K. (1991). Levels of information and contributions to public goods. *Social Forces*, *70*(1), 107–124.

Shanley, J., & Grossman, P. J. (2007). Paradise to parking lots: Creation versus maintenance of a public good. *The Journal of Socio-Economics*, *36*(4), 523–536.

Solow, J. L., & Kirkwood, N. (2002). Group identity and gender in public goods experiments. *Journal of Economic Behavior & Organization*, *48*(4), 403–412.

Stoddard, B., Walker, J. M., & Williams, A. (2014). Allocating a voluntarily provided common-property resource: An experimental examination. *Journal of Economic Behavior & Organization*, *101*, 141–155.

Stoddard, B. V., et al. (2014). Probabilistic production of a public good. *Economics Bulletin*, *34*(4), 2427–2442.

Sutter, M., Haigner, S., & Kocher, M. G. (2010). Choosing the carrot or the stick? endogenous institutional choice in social dilemma situations. *The Review of Economic Studies*, *77*(4), 1540–1566.

van Soest, D., Stoop, J., & Vyrastekova, J. (2016). Toward a delineation of the circumstances in which cooperation can be sustained in environmental and resource problems. *Journal of Environmental Economics and Management*, *77*, 1–13.

Vyrastekova, J., Funaki, Y., & Takeuchi, A. (2011). Sanctioning as a social norm: Expectations of non-strategic sanctioning in a public goods game experiment. *The Journal of Socio-Economics*, *40*(6), 919–928.

Weimann, J. (1994). Individual behaviour in a free riding experiment. *Journal of Public Economics*, *54*(2), 185–200.

Xiao, E., & Houser, D. (2011). Punish in public. *Journal of Public Economics*, *95*(7), 1006–1017.
