# Supplementary material for: Transparency and cooperation in repeated dilemma games: a meta study
Source: Exp Econ. 2017 Feb 24;20(4):755–71. doi: 10.1007/s10683-017-9517-4 (PMC5665965; doi:10.1007/s10683-017-9517-4)
Supplement: Supplementary file 2 — Supplementary material 2 (pdf 90 KB) [file 10683_2017_9517_MOESM2_ESM.pdf]

S2 Included Studies Oligopoly Experiments

Article	F.aggchoice	F.indichoice	F.indipayoff	F.ownpayoff
Apesteguia et al. (2010)		X	X	X
Coq and Orzen (2006)	X			X
Davis et al. (2003)	X			
Davis et al. (2010)		X		X
Davis (2002)	X	Y	Y	X
Davis (2011)	X			X
Fouraker and Siegel (1963)	Y			X
Gallego (1998)		X		X
Gürerk and Selten (2012)		X	X	X
Huck et al. (1999)		Y	Y	X
Huck et al. (2000)	X	Y	Y	X
Huck et al. (2002)	X			X
Huck et al. (2004)	Y			X
Offerman et al. (2002)	X	Y	Y	X
Roux and Thöni (2015)	X			X
Sonntag and Zizzo (2014)	X			X
Vriend and Bosch-Domenech (2003)		X	Y	X
Waichman et al. (2010)	X			X

Notes: X means used in all treatments, Y means used in some treatments only, no symbol means feedback not given, and n.a. means data is missing. F.aggchoice refers to aggregate feedback about choices in one's group, F.indichoice to feedback about each group member's choice, F.indipayoffs to feedback about each group member's payoff, and F.ownpayoff to feedback about one's own payoff.

List of Oligopoly Studies

Apesteguia, J., Huck, S., Oechssler, J., & Weidenholzer, S. (2010). Imitation and the evolution of Walrasian behavior: Theoretically fragile but behaviorally robust. *Journal of Economic Theory*, *145*(5), 1603–1617.

Bosch-Domènech, A., & Vriend, N. J. (2003). Imitation of successful behaviour in Cournot markets. *The Economic Journal*, *113*(487), 495–524.

Davis, D. (2011). Behavioral convergence properties of Cournot and Bertrand markets: an experimental analysis. *Journal of Economic Behavior & Organization*, *80*(3), 443–458.

Davis, D., Korenok, O., & Reilly, R. (2010). Cooperation without coordination: signaling, types and tacit collusion in laboratory oligopolies. *Experimental Economics*, *13*(1), 45–65.

Davis, D. D. (2002). Strategic interactions, market information and predicting the effects of mergers in differentiated product markets. *International Journal of Industrial Organization*, *20*(9), 1277–1312.

Davis, D. D., Reilly, R. J., & Wilson, B. J. (2003). Cost structures and Nash play in repeated Cournot games. *Experimental Economics*, *6*(2), 209–226.

Fouraker, L., & Siegel, S. (1963). *Bargaining behavior.* New York: McGraw-Hill.

Gallego, A. G. (1998). Oligopoly experimentation of learning with simulated markets. *Journal of Economic Behavior & Organization*, *35*(3), 333–355.

Gürerk, Ö., & Selten, R. (2012). The effect of payoff tables on experimental oligopoly behavior. *Experimental Economics*, *15*(3), 499–509.

Huck, S., Normann, H.-T., & Oechssler, J. (1999). Learning in Cournot oligopoly–An experiment. *The Economic Journal*, *109*(454), 80–95.

Huck, S., Normann, H.-T., & Oechssler, J. (2000). Does information about competitors actions increase or decrease competition in experimental oligopoly markets? *International Journal of Industrial Organization*, *18*(1), 39–57.

Huck, S., Normann, H.-T., & Oechssler, J. (2002). Stability of the Cournot process–experimental evidence. *International Journal of Game Theory*, *31*(1), 123–136.

Huck, S., Normann, H.-T., & Oechssler, J. (2004). Two are few and four are many: number effects in experimental oligopolies. *Journal of Economic Behavior & Organization*, *53*(4), 435–446.

Le Coq, C., & Orzen, H. (2006). Do forward markets enhance competition? Experimental evidence. *Journal of Economic Behavior & Organization*, *61*(3), 415–431.

Offerman, T., Potters, J., & Sonnemans, J. (2002). Imitation and belief learning in an oligopoly experiment. *The Review of Economic Studies*, *69*(4), 973–997.

Roux, C., & Thöni, C. (2015). Collusion among many firms: The disciplinary power of targeted punishment. *Journal of Economic Behavior & Organization*, *116*, 83–93.

Sonntag, A., & Zizzo, D. J. (2015). Institutional authority and collusion. *Southern Economic Journal*, *82*(1), 13–37.

Waichman, I., Requate, T., & Siang, C. K. (2010). A Cournot experiment with managers and students: evidence from Germany and Malaysia. *The BE Journal of Economic Analysis & Policy*, *10*(1), Article 30.
