# Supplementary material for: Transparency and cooperation in repeated dilemma games: a meta study
Source: Exp Econ. 2017 Feb 24;20(4):755–71. doi: 10.1007/s10683-017-9517-4 (PMC5665965; doi:10.1007/s10683-017-9517-4)
Supplement: Supplementary file 3 — Supplementary material 3 (pdf 101 KB) [file 10683_2017_9517_MOESM3_ESM.pdf]

S3 Supplementary Tables

Table S1: Unweighted Regression Results Public Goods

Dep. var.: Share of endowment contributed								
	(1)	(2)	(3)	(4)	(5)	(6)	(7)	(8)
F.indichoice	.015				.025	.037	.030	.048*
	(.563)				(.400)	(.170)	(.323)	(.080)
F.aggchoice		-.012			-.021	-.023	-.023	-.026
		(.711)			(.578)	(.520)	(.551)	(.456)
F.indipayoffs			-.059		-.082*	-.117***	-.083*	-.121***
			(.156)		(.087)	(.009)	(.083)	(.006)
F.ownpayoff				-.010	.000	.050	.004	.061
				(.792)	(.995)	(.187)	(.922)	(.107)
MPCR						.427***		.458***
						(.000)		(.000)
Group size							.002	.004*
							(.388)	(.057)
Constant	.395***	.411***	.407***	.408***	.414***	.173***	.398***	.123*
	(.000)	(.000)	(.000)	(.000)	(.000)	(.009)	(.000)	(.078)
R^2	.003	.001	.018	.001	.031	.211	.038	.237
Adj. R^2	-.006	-.008	.009	-.008	-.005	.174	-.007	.194
N	116	115	116	114	113	113	113	113

Notes: The table reports results from unweighted linear regressions based on data from public goods experiments (p-values in parentheses). Stars ***, ** or * indicate that the effect of the variable is statistically significant at the 1%, 5% or 10% level, respectively. F.aggchoice refers to aggregate feedback about choices in one's group, F.indichoice to feedback about each group member's choice, F.indipayoffs to feedback about each group member's payoff, and F.ownpayoff to feedback about one's own payoff.

Table S2: Unweighted Regression Results Public Goods with MPCR <0.7

Dep. var.: Share of endowment contributed								
	(1)	(2)	(3)	(4)	(5)	(6)	(7)	(8)
F.indichoice	.018				.040	.037	.045	.046
	(.503)				(.184)	(.210)	(.148)	(.121)
F.aggchoice		-.001			-.004	-.016	-.005	-.020
		(.985)			(.926)	(.682)	(.902)	(.592)
F.indipayoffs			-.122***		-.138***	-.146***	-.138***	-.149***
			(.010)		(.007)	(.003)	(.007)	(.003)
F.ownpayoff				-.027	-.017	.034	-.013	.052
				(.495)	(.671)	(.419)	(.743)	(.235)
MPCR						.364***		.430***
						(.007)		(.002)
Group size							.002	.004*
							(.435)	(.095)
Constant	.379***	.388***	.398***	.409***	.397***	.209**	.382***	.143
	(.000)	(.000)	(.000)	(.000)	(.000)	(.013)	(.000)	(.119)
R^2	.004	.000	.064	.005	.085	.154	.091	.179
Adj. R^2	-.005	-.010	.055	-.005	.047	.109	.043	.126
N	104	103	104	102	101	101	101	101

Notes: The table reports results from unweighted linear regressions based on data from public goods experiments with MPCR < 0.7 (p-values in parentheses). Stars ***, ** or * indicate that the effect of the variable is statistically significant at the 1%, 5% or 10% level, respectively. F.aggchoice refers to aggregate feedback about choices in one's group, F.indichoice to feedback about each group member's choice, F.indipayoffs to feedback about each group member's payoff, and F.ownpayoff to feedback about one's own payoff.

Table S3: Unweighted Regression Results Oligopoly

Dep. var.: Degree of collusion								
	(1)	(2)	(3)	(4)	(5)	(6)	(7)	(8)
F.indichoice	-.071				.873***	.709***	.244	.711***
	(.624)				(.000)	(.001)	(.289)	(.001)
F.aggchoice		.175			.429***	.422***	.448***	.424***
		(.240)			(.009)	(.005)	(.002)	(.006)
F.indipayoffs			-.530***		-1.039***	-.848***	-.448**	-.853***
			(.000)		(.000)	(.000)	(.030)	(.000)
F.ownpayoff				-.095	-.032	-.120	.581	-.110
				(.854)	(.936)	(.740)	(.214)	(.765)
Complements						.393***	.388***	.395***
						(.002)	(.006)	(.003)
'Friedman' index							.295**	
							(.041)	
Group size								-.011
								(.848)
Constant	-.121	-.265**	-.004	-.060	-.489	-.482	-1.393**	-.451
	(.210)	(.029)	(.954)	(.906)	(.246)	(.210)	(.013)	(.284)
R^2	.005	.029	.229	.001	.458	.562	.514	.562
Adj. R^2	-.016	.008	.213	-.020	.410	.512	.439	.501
N	50	50	50	50	50	50	46	50

Notes: The table reports results from unweighted linear regressions based on data from oligopoly experiments. The numbers of independent observations by unit of observation (treatment) are used as weights (p-values in parentheses). Stars ***, ** or * indicate that the effect of the variable is statistically significant at the 1%, 5% or 10% level, respectively. F.aggchoice refers to aggregate feedback about choices in one's group, F.indichoice to feedback about each group member's choice, F.indipayoffs to feedback about each group member's payoff, and F.ownpayoff to feedback about one's own payoff.

Table S4: Weighted Regression Public Goods Reduced Sample

Dep. var.: Share of endowment contributed					
	(1)	(2)	(3)	(4)	(5)
F.indichoice	.030				.043
	(.271)				(.156)
F.aggchoice		-.001			-.001
		(.982)			(.973)
F.indipayoffs			-.033		-.054
			(.492)		(.345)
F.ownpayoff				.001	.001
				(.984)	(.987)
Constant	.406***	.423***	.425***	.419***	.402***
	(.000)	(.000)	(.000)	(.000)	(.000)
R^2	.011	.000	.004	.000	.026
Adj. R^2	.002	-.009	-.005	-.009	-.013
N	110	109	110	108	107

Notes: The table reports results from linear regressions based on data from public goods experiments. The numbers of independent observations by unit of observation (treatment) are used as weights (p-values in parentheses). Stars ***, ** or * indicate that the effect of the variable is statistically significant at the 1%, 5% or 10% level, respectively. F.aggchoice refers to aggregate feedback about choices in one's group, F.indichoice to feedback about each group member's choice, F.indipayoffs to feedback about each group member's payoff, and F.ownpayoff to feedback about one's own payoff.

Table S5: Weighted Regression Oligopoly Reduced Sample

Dep. var.: Degree of collusion					
	(1)	(2)	(3)	(4)	(5)
F.indichoice	-.184				
	(.185)				
F.aggchoice		.184			-.525***
		(.185)			(.000)
F.indipayoffs			-.602***		- 1.051***
			(.000)		(.000)
F.ownpayoff				-.146	-.085
				(.715)	(.665)
Constant	-.141*	-.325***	-.069	-.060	.465**
	(.079)	(.007)	(.176)	(.879)	(.042)
R^2	.067	.067	.556	.005	.783
Adj. R^2	.031	.031	.538	-.033	.756
N	28	28	28	28	28

Notes: The table reports results from linear regressions based on data from oligopoly experiments. The numbers of independent observations by unit of observation (treatment) are used as weights (p-values in parentheses). Stars ***, ** or * indicate that the effect of the variable is statistically significant at the 1%, 5% or 10% level, respectively. F.aggchoice refers to aggregate feedback about choices in one's group, F.indichoice to feedback about each group member's choice, F.indipayoffs to feedback about each group member's payoff, and F.ownpayoff to feedback about one's own payoff.
